# The Cook County Insane Asylum

**Published:** 1886-03

**Authors:** 


THE COOK COUNTY INSANE ASYLUM.

The report of the STATE BOARD OF CHARITIES, recently published, regarding the abuses at the Cook County Insane Asylum, contains some important information and merits careful reading and thought. The first portion of the report deals with evils and abuses as they were found to exist, and were brought out by the testimony given before the committee. Political considerations were found to largely govern the appointment of employés. Conflicts among officers, lax discipline and insubordination are among the evils mentioned. It was found that supplies cannot be easily and quickly procured, and that the patients suffered various "inconveniences" in consequence. This latter statement refers to the sworn testimony of the assistant physician, that at one time over fifty patients suffered from scurvy, and as the result deaths were common. It will be readily admitted that these results were at least "inconvenient" to the patients.

It is not, however, so much with the findings of the committee, which are in the main correct and in accordance with the testimony, as with the remedies suggested, that we take issue.

First, the suggestion that the State take charge of the institution, is disposed of by the Board as impracticable at the present time, because the proximity of the county house and the small size of the county farm are insuperable objections. The facts that the institution has no adequate water-supply and but very imperfect drainage, are much more vital considerations, in our opinion.

Second, that the County Commissioners should place the care of the institution in the hands of a Board of Trustees, who would organize and govern the place substantially as State institutions are governed is recommended. This admirable plan having succeeded so well in many of our State institutions, it is but natural that it should have the earnest support of the State Board of Charities. Unfortunately the problem is quite different. With the State these matters are regulated by the statute, and the legislature cannot interfere except by an amendment to the organic law. The constitution directs that the affairs of Cook county shall be managed by a board of fifteen commissioners. Their power is absolute: they appropriate funds, purchase supplies, pay the bills, audit their accounts, and finally, but not least, appoint the grand jury, the only body which has authority to impeach them. Any limitation of their powers is purely voluntary and may be abridged or altered at any time only by themselves. Is it to be supposed that all of the Commissioners will voluntarily surrender their control of our largest public charity to the care of a few men whose only aim would be the benefit of the unfortunate? How long would men of character and integrity be found willing to retain such positions, after influence was brought to bear in the interests of "the party?"

Third, that the State Board of Charities assume the relation of trustees as outlined in the above plan. This is decidedly the best suggestion, as the State Board is, by its character and manner of its appointment, beyond the reach of any local influence. But this plan is open to the objection that so much control as is surrendered is only voluntary, and may be entirely abrogated by a simple majority vote at any meeting. Any dispute that may arise could be easily settled by the County Commissioners withholding the appropriation, when the management must come to terms or give up their control.

The fact remains that ever since the establishment of the "Cook County Crazy-House," over thirty years ago, the management of that charity has been a disgrace. It would seem as if the only remedy is for the State to establish one or more additional hospitals, and to take charge of the insane now in the county house. The facts regarding the Cook county insane are true of most of all the insane now in county receptacles. The State cares for at least seventy per centum, and there is no reason why its care should not be extended to all.

County provision for the insane has always proved from 30 to 50 per centum more expensive than State care. The annual saving to the tax-payers of this county would build a new hospital in a few years, besides conferring an inestimable boon upon a neglected and unfortunate class of our community.

THE MAY MEETING OF THE ILLINOIS STATE MEDICAL SOCIETY.

The eminently successful character of the Eightieth Annual Meeting of the MEDICAL SOCIETY OF THE STATE OF NEW YORK, recently held in Albany, places in conspicuous and unpleasant relief the usual features of the annual gatherings of the ILLINOIS STATE MEDICAL SOCIETY. The papers read in Albany were in the large majority of cases of great practical interest, and founded upon personal observation and experiment. There was a refreshing absence of reports of chairmen of Sections, embodying the progress of medical science in their respective departments. These reports, demanded by the constitution of our State Society, degenerate only too frequently into compilations from the various American medical journals, which every practitioner receives. Even admitting that the report be prepared conscientiously and in conformity with the design of the framers of the constitution, the space of twenty minutes,—the period of time allotted to each chairman,—is inadequate to the demands of the occasion. But the necessity for systematic reports, on progress of the medical science, does not exist at the present day, as it did even a few years ago. With the appearance ot the *Index Rerum*, the various *Retrospects*, *Digests*, *anelectics*, and *special departments* in our leading periodicals, has not the preparation of laborious *résumés* of novelties become a work of supererogation? Would not the critical discussion of a single topic, or the narration and analysis of an instructive case prove of greater interest and importance under existing conditions?

At the Chicago meeting, nearly two years ago, S. Walter Hay presented the following preamble and resolutions:

WHEREAS, In the preamble to the resolution determining the formation of the State Medical Society it is set forth, as one of the objects of the Society, that "it shall supply more efficient means than have hitherto been available for cultivating and advancing medical knowledge, etc.," therefore, be it

*Resolved*, That a prize of $100.00 be offered by the Society for the best report of original investigations into the Etiology, Pathology and Treatment of Diphtheria.

That a second prize of $100.00 be offered for the best tabulated report of not less than ten cases of any form of disease under the professional care of the reporter.

The resolutions were adopted, and Drs. Walter Hay, H. A. Johnson, and John E. Owens were appointed a Committee on "Prize Essays." This Committee made no report at Springfield last year, for the reason that there were no candidates for the honors. The sum of one hundred dollars, of course, is entirely inadequate to the expenses involved in any investigation of Diphtheria, but the second prize ought to have enlisted interest. As informed at present, the Society continues to offer both prizes under the same conditions.

Probably no state west of the Alleghanies is of as great interest to the general profession of the country as Illinois. This fact is probably due to the important acts in medical legislation secured within recent years, under great obstacles, by the determined and united efforts of the local profession. Much remains to be done in this important field. The hands of the members of the State Board of Health must be upheld in the prosecution of their arduous labors.

Fortunately the Demonstrators' Association no longer requires aid in securing anatomical material. But then, legislation with reference to the insane is in a very chaotic state, as recent developments in the care of this unfortunate class in Cook County abundantly demonstrate.

